# Prevalence of Chronic Myocardial Ischemia and Gastrointestinal Bleeding Risk in Patients With Chronic Kidney Disease Undergoing Dual Antiplatelet Therapy

**DOI:** 10.7759/cureus.95538

**Published:** 2025-10-27

**Authors:** Muhammad Mughees Israr, Talha Aftab, Pardeep Sainani, Ramsha Zuberi, Bahar Liza Fredie, Muhammad Tayyab, Adeel Ur Rehman, Sawera Nadeem

**Affiliations:** 1 Medicine, Fatima Memorial Hospital College of Medicine and Dentistry, Lahore, PAK; 2 Medicine, Services Hospital, Lahore, PAK; 3 Nephrology, Bahria University Health Sciences Campus, Karachi, PAK; 4 Medicine, Dow University of Health and Sciences, Karachi, PAK; 5 Research, People’s Medical College Hospital, Nawabshah, PAK; 6 Cardiology, Azra Naheed Medical College, Lahore, PAK; 7 Neurosurgery, Punjab Institute of Neurosciences, Lahore, PAK; 8 General Medicine, Hazrat Barri Imam Sarkar (HBS) General Hospital, Islamabad, PAK

**Keywords:** antiplatelet, gastrointestinal pathology, patients satisfaction, risk factors, therapy

## Abstract

Background

Chronic kidney disease (CKD) is strongly associated with an increased risk of cardiovascular morbidity and mortality, with chronic myocardial ischemia being a frequent complication.

Objective

The objective of this study is to determine the prevalence of chronic myocardial ischemia and evaluate the gastrointestinal bleeding risk in CKD patients receiving dual antiplatelet therapy.

Methods

This cross-sectional observational study was conducted at the Dow University of Health and Sciences in Karachi between January and July 2025. A total of 180 patients with diagnosed CKD who were undergoing dual antiplatelet therapy were chosen for the study. Clinical assessment, laboratory parameters, and electrocardiographic findings were used to evaluate chronic myocardial ischemia. Gastrointestinal bleeding risk was assessed through clinical history, endoscopic evaluation (when indicated), and validated bleeding risk scores.

Results

Among 180 CKD patients, 74 (41.1%) demonstrated evidence of chronic myocardial ischemia on ECG and clinical evaluation. Gastrointestinal bleeding events were documented in 32 (17.8%) patients, while an additional 46 (25.6%) were classified as high risk for bleeding based on clinical and endoscopic assessment. Patients in advanced CKD stages (Stage 4 and 5, n=110) had a significantly higher prevalence of myocardial ischemia (57/110, 51.8%) and gastrointestinal bleeding risk (31/110, 28.2%) compared to those in earlier stage CKD (Stage 3, n=70; ischemia 20/70, 28.6%; bleeding risk 12/70, 17.1%) (p<0.05).

Conclusion

Chronic myocardial ischemia and gastrointestinal bleeding risk are both highly prevalent in CKD patients undergoing dual antiplatelet therapy. Nearly half of the patients exhibited ischemic burden, while one-fifth experienced bleeding events.

## Introduction

Dual antiplatelet therapy (DAPT) plays a vital role in the management of acute coronary syndrome (ACS) [1] but predisposes patients to gastrointestinal (GI) bleeding, and the incidence of such events is 3.9% within 30 days [2]. Cardiovascular disease and chronic kidney disease (CKD) co-exist in many patients, with CKD being present in 33%-50% of patients with an acute myocardial infarction (AMI) [1]. CKD leads to abnormal homeostasis and other comorbid conditions that increase the risk of bleeding in patients with CKD [3]. The causes of its increased prevalence continue to soar with the aging populations and the growing occurrences of diabetes and hypertension rates, the two most common causes of CKD. In addition to the progressive decline of renal function, CKD has started to be understood as a systemic tendency that dramatically magnifies susceptibility to heart and vascular complications [4]. Cardiovascular disease is the leading cause of death among patients with CKD, and cardiac ischemia is one of the most common sequelae of chronic myocardial ischemia. CKD-related risk factors, including uremic toxins, vascular calcification, and chronic inflammation, in combination with the traditional risk factors like hypertension, diabetes, and dyslipidemia, accelerate atherosclerosis and lead to early onset and nadir manifestations of ischemic heart disease [5]. Notably, due to atypical or even silent ischemia, the prognoses are aggravated in CKD patients who are more difficult to manually diagnose timely manner than those whose renal condition remained stable [6]. In CKD patients, myocardial ischemia is multifactorial in occurrence. Deliberately reduced endothelial elimination of uremic toxins and ongoing oxidative stress damage endothelial performance, lessening nitric oxide levels and increasing artery rigidity. Moreover, anemia and calcium-phosphorus metabolism disorders cause an increase in the load on the heart and accelerate vascular calcification, which additionally deteriorates the ischemic load [7]. Such pathophysiologies develop into a cardiovascular high-risk phenotype among patients with CKD that justifies stringent preventive and treatment efforts. DAPT, which includes the use of aspirin and a P2Y12 inhibitor, including clopidogrel, prasugrel, or ticagrelor, is regularly used to minimize thrombotic events in the case of patients with acute coronary syndromes or those who are having percutaneous coronary intervention [8]. Its role in decreasing ischemic events has been proven in the general population, but in the CKD population, any potential benefits are counterbalanced by a greatly increased risk of bleeding manifestations [9].

One of the most threatening side effects of DAPT refers to gastrointestinal (GI) bleeding, and CKD patients are overrepresented. These predisposing factors are uremia-induced platelet dysfunction, a change in coagulation pathways, inefficient mucosal healing, and the frequent co-medication of anticoagulants or gastrotoxic drugs contribute to bleeding [10]. Moreover, CKD patients with GI bleeding are not only more common but also more severe, more likely to result in hospitalization, the need to transfuse blood, and, in some instances, the need to prematurely stop DAPT. However, this discontinuation, in turn, may be harmful and trigger a higher probability of a myocardial infarction, stent thrombosis, or mortality [11]. As a result, CKD patients face a major challenge in maintaining this delicate balance between ischemic protection and bleeding prevention. Within a maximum follow-up period of one to two years, the majority of Western studies have reported GI bleed rates of 0.7%-1.3% for aspirin and 1.2%-2% for aspirin+clopidogrel combination (DAPT) [12]. Data on long-term (>10 years) incidence of GI bleed are sparse, reported as 2.1% in men after 14 years [13] and 1.75% in women after 24 years of aspirin use [14]. We hypothesized that the prevalence of both chronic myocardial ischemia and gastrointestinal bleeding risk increase with advancing CKD stage. These conditions may coexist due to overlapping mechanisms, such as vascular dysfunction and platelet hyperreactivity, further exacerbated by DAPT exposure. Understanding these combined risks is crucial for optimizing treatment strategies [15].

Objective

The objective of this study is to determine the prevalence of chronic myocardial ischemia in CKD patients and evaluate the gastrointestinal bleeding risk in CKD patients receiving dual antiplatelet therapy.

## Materials and methods

This cross-sectional observational study was conducted at Dow University of Health and Sciences between January and July 2025. A total of 180 patients diagnosed with CKD who were on DAPT were included in the study. The sample size was calculated to achieve adequate statistical power to detect differences in prevalence rates with a 95% confidence interval and 5% margin of error. Patients were selected using a non-probability consecutive sampling method. The study protocol was approved by the Institutional Ethical Review Committee with ethical approval number DUHS/IRB/ERC/3421/2024. Written informed consent was obtained from all participants before enrollment.

Data collection procedure

Patient demographics, clinical history, and CKD stage of all the patients were recorded. Chronic myocardial ischemia was assessed through clinical evaluation, ECG findings (ST-T changes, Q-waves), and review of previous angiographic or stress test results when available. Gastrointestinal bleeding risk was evaluated through clinical history of hematemesis, melena, or hematochezia, along with laboratory assessment (hemoglobin drop >2 g/dl), stool occult blood testing, and endoscopic findings where indicated. A validated bleeding risk score (such as Hypertension, Abnormal Renal/Liver Function, Stroke, Bleeding History or Predisposition, Labile INR, Elderly, Drugs/Alcohol Concomitant Score (HAS-BLED)) was also applied to stratify patients into low, moderate, and high risk (https://www.mdcalc.com/calc/807/has-bled-score-major-bleeding-risk).

Data analysis

All collected data were entered and analyzed using IBM SPSS Statistics, version 26.0 (IBM Corp, Armonk, NY). Continuous variables such as age and laboratory values were presented as mean±standard deviation (SD), while categorical variables such as presence of ischemia and gastrointestinal bleeding were expressed as frequencies and percentages. Chi-square test was applied to evaluate associations between categorical variables, and an independent t-test for continuous data. A p-value of <0.05 was considered statistically significant.

## Results

A total of 180 patients with CKD receiving DAPT were included in the study. The mean age of participants was 58.6±11.4 years, with 112 (62.2%) men and 68 (37.8%) women. Most patients were in advanced stages of CKD, with 38.9% in stage 3, 33.9% in stage 4, and 27.2% in stage 5. Hypertension (74.4%) and diabetes mellitus (63.9%) were the most prevalent comorbidities, while dyslipidemia (26.7%) and smoking (23.3%) were less frequent. Among the 32 patients who experienced GI bleeding, melena was the predominant presentation (62.5%), followed by hematemesis (28.1%) and hematochezia (9.4%) (Table 1).

**Table 1:** Baseline Demographic and Clinical Characteristics of Patients (n=180)

Variable	Mean±SD / n (%)
Age (years)	58.6±11.4
Gender	
Male	112 (62.2%)
Female	68 (37.8%)
CKD Stage	
Stage 3	70 (38.9%)
Stage 4	61 (33.9%)
Stage 5	49 (27.2%)
Comorbidities	
Hypertension	134 (74.4%)
Diabetes mellitus	115 (63.9%)
Dyslipidemia	48 (26.7%)
Smoking	42 (23.3%)
Clinical Presentation	(n=32 with GI bleed)
Melena	20 (62.5%)
Hematemesis	9 (28.1%)
Hematochezia	3 (9.4%)

**Figure 1:**
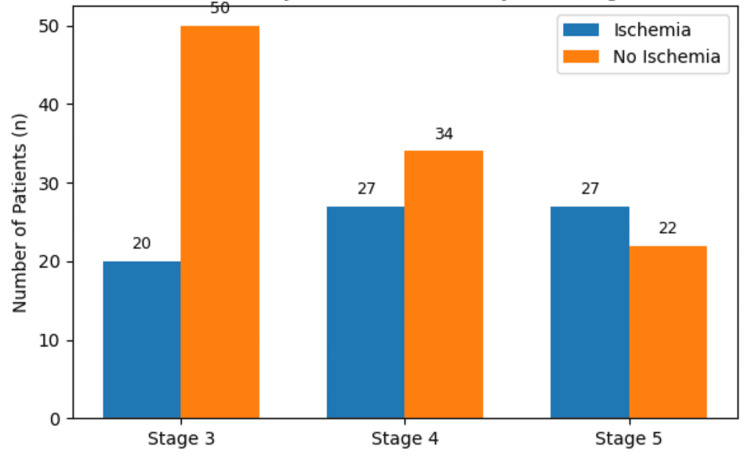
Chronic Myocardial Ischemia by CKD stages

Ischemia was present in 74 (41.1%) overall, but prevalence rose sharply with CKD progression: 20 (28.6%) in Stage 3, 27 (44.3%) in Stage 4, and 27 (55.1%) in Stage 5. The between-stage difference was statistically significant (χ², p=0.01), indicating a clear gradient of ischemic burden with declining renal function (Table 2, Figure 1).

**Table 2:** Prevalence of Chronic Myocardial Ischemia by CKD Stage (n=180) *Chi-square test applied.

CKD Stage	Total Patients	Ischemia Present, n (%)	p-value
Stage 3	70	20 (28.6)	0.01*
Stage 4	61	27 (44.3)	
Stage 5	49	27 (55.1)	
Total	180	74 (41.1)	

Out of 74 patients with ischemia, 21 (28.4%) experienced GI bleeding or were classified as high-risk, compared with only 12 (11.3%) among the 106 patients without ischemia. Overall, 33 of 180 patients (18.3%) demonstrated bleeding events or high-risk status. The difference between ischemic and non-ischemic groups was statistically significant (p = 0.01) (Table 3).

**Table 3:** Association of Myocardial Ischemia with Gastrointestinal Bleeding Risk (n=180) *Chi-square test applied.

Group	Total Patients	GI Bleeding /High Risk, n (%)	p-value
Ischemia Present	74	21 (28.4)	0.01*
No Ischemia	106	12 (11.3)	
Total	180	33 (18.3)	

**Figure 2:**
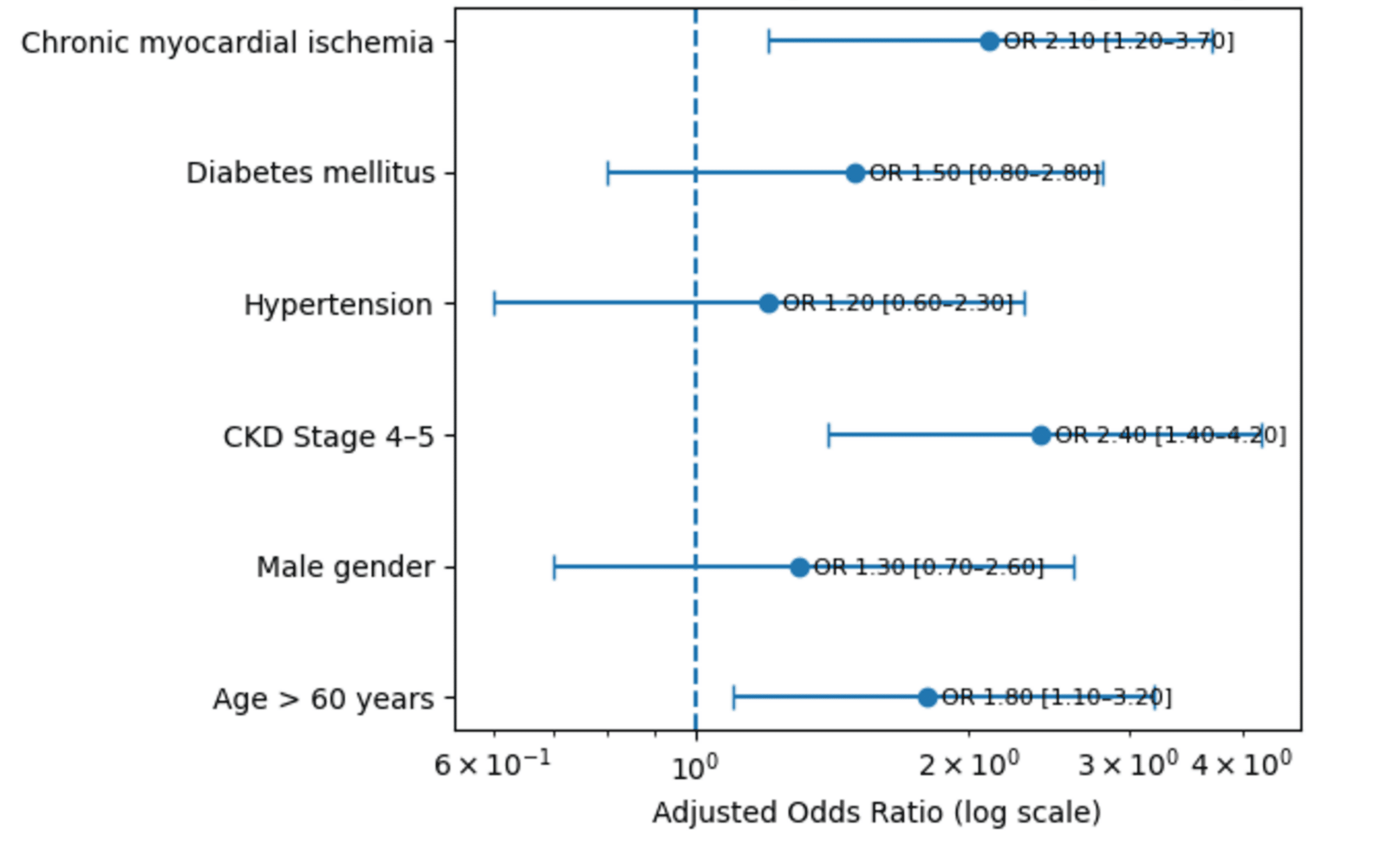
Predictors of GI Bleeding

After adjustment, three predictors independently signaled higher bleeding risk: CKD Stage 4-5 (adjusted odds ratio (aOR) 2.4, 95% CI 1.4-4.2; p=0.01), chronic myocardial ischemia (aOR 2.1, 95% CI 1.2-3.7; p=0.01), and age >60 years (aOR 1.8, 95% CI 1.1-3.2; p=0.04). Male sex, hypertension, and diabetes were not significant in the adjusted model (Table 4, Figure 2).

**Table 4:** Multivariate Logistic Regression Analysis of Predictors of Gastrointestinal Bleeding (n = 180) *Logistic regression model adjusted for age, gender, comorbidities, and CKD stage. OR = Odds Ratio; CI = Confidence Interval.

Predictor Variable	Adjusted OR	95% CI	p-value
Age > 60 years	1.8	1.1 – 3.2	0.04*
Male gender	1.3	0.7 – 2.6	0.29
CKD Stage 4–5	2.4	1.4 – 4.2	0.01*
Hypertension	1.2	0.6 – 2.3	0.37
Diabetes mellitus	1.5	0.8 – 2.8	0.18
Chronic myocardial ischemia	2.1	1.2 – 3.7	0.01*

## Discussion

This study investigated the prevalence of chronic myocardial ischemia and GI bleeding risk among patients with CKD receiving DAPT. The results indicate that ischemic burden and bleeding risk are very common in this population and 41.1% of the patients show the signs of chronic myocardial ischemia, with 17.8% of the cases having overt GI bleeding. Another 25.6% were found to be at high risk of bleeding, which is indicative of the complex mechanism of interaction between cardiovascular protection and hemorrhagic events in CKD patients who use DAPT. The overall prevalence of chronic myocardial ischemia in this cohort is similar to prior findings, which have indicated that CKD is a strong independent risk factor of coronary artery disease [16]. Known pathophysiological mechanisms and validated to cause ischemic burden in CKD patients include vascular calcification, vascular endothelial dysfunction, and chronic inflammation. The incidence of ischemia did rise as renal dysfunction deteriorated in this analysis and exceeded 50% in Stage 5 CKD. This corroborates the idea that severe kidney dysfunction is a driving factor of atherosclerotic disease progression and the relevance of close cardiovascular risk monitoring in end-stage CKD [17].

Likewise, the burden of GI bleeding presented in the current study is consistent with the previous literature as there have been consistent findings on an increased bleeding risk in CKD patients. One in five patients experienced severe GI bleeding, while over 40% were at high risk of bleeding or showing related symptoms [18]. The most prevalent clinical manifestation was melena, indicative of involvement of the upper GI tract that is characteristic of patients on aspirin-type antiplatelet therapy. Notably, the risk of bleeding was much higher among patients with relatively advanced stages of CKD (Stages 4 and 5), as opposed to patients with Stage 3 [19]. This observation can be explained by an even more severe dysfunction of platelets, fragility of mucosae, and intake of gastrotoxic drugs in advanced disease.

Particularly interesting is the relation between risk of GI bleeding and chronic myocardial ischemia. Here, 28.4% of the ischemic patients also exhibited bleeding risk, and only 11.3% in the non-ischemic category. Such a dual burden illustrates a therapeutic dilemma of DAPT use in patients with CKD: the ones with the greatest ischemic risk are also the most susceptible to bleeding. The existence of such a dilemma, commonly referred to as the ischemia bleeding trade-off, has been emphasized in earlier studies and makes clinical decision-making in some high-risk individuals, including CKD, challenging [20]. Multivariate analysis also attested that more severe stages of CKD and the occurrence of ischemia were all independent predictors of GI bleeding. The risks of bleeding were also higher in patients 60 years and above in keeping with the available literature that found age as a significant predictor of hemorrhagic complications. These results underline the importance of selecting a patient carefully and making therapy personalized. Although DAPT represents a mainstay in the prevention of further ischemic complications, DAPT is a complex intervention in CKD that requires active bleeding risk reduction measures, so gastroprotective treatment is recommended along with close response to hemoglobin changes [21]. The study has a number of implications in a clinical sense. First, risk stratification of ischemic risk in patients with CKD cannot be narrowed down to symptom-oriented assessment, with unusual manifestations, potentially resulting in underestimation of myocardial ischemia. Routine electrocardiography and, where possible, non-invasive testing of ischemia may be of assistance in earlier detection.

Limitations

The limitations of this study must be acknowledged. Being cross-sectional in design, it cannot establish causal relationships between CKD progression, ischemia, and bleeding risk. Endoscopic confirmation was not feasible for all patients, and subclinical bleeding may have been underestimated. Additionally, the use of a single-center cohort may limit generalizability to broader populations. Despite these limitations, the study provides important insights into the dual burden of ischemic and bleeding complications in CKD patients receiving DAPT.

## Conclusions

It is concluded that chronic myocardial ischemia and GI bleeding risk are both highly prevalent in patients with CKD undergoing DAPT. In this study, more than two-fifths of patients demonstrated evidence of ischemia, while nearly one-fifth experienced overt gastrointestinal bleeding and an additional one-fourth were classified as high-risk. Advanced CKD stages were significantly associated with both higher ischemic burden and increased bleeding risk, and patients with ischemia were more likely to also develop bleeding complications. These findings highlight the therapeutic challenge of balancing ischemic protection with bleeding prevention in this vulnerable population. Individualized risk stratification, close monitoring, and proactive preventive strategies such as gastroprotective therapy are essential to optimize outcomes and reduce complications in CKD patients requiring dual antiplatelet therapy.
